# Scoping Review of the Psychological Effects of Gender-Based Violence on Children

**DOI:** 10.3390/children12091277

**Published:** 2025-09-22

**Authors:** Maria Rodriguez Rodriguez, Diego Gomez-Baya

**Affiliations:** Department of Social, Developmental and Educational Psychology, University of Huelva, 21071 Huelva, Spain; maria.rodriguez8@alu.uhu.es

**Keywords:** gender-based violence, childhood, psychological consequences, externalizing symptoms, internalizing symptoms

## Abstract

The lack of acknowledgment of children as victims of gender-based violence hinders the support they receive. This study aimed to identify the psychological consequences of children’s exposure to gender-based violence and gaps in knowledge. This work used a scoping review approach, based on the PRISMA quality criteria. The search was conducted in the 14 databases included in the Web of Science platform. A total of 13 open-access articles published in English between 2015 and 2025 that focus on gender-based violence psychological consequences in children met the inclusion criteria. The results of the review indicate that gender violence has significant negative psychological, emotional, and social effects on children exposed to it. Thus, symptoms of internalizing, externalizing, and post-traumatic stress disorder may appear. Additionally, there is a high probability of experiencing difficulties in school, interpersonal relationships, and identity development. These effects may have long-term consequences affecting well-being and development later in life. It is crucial to recognize children as direct and significant victims of gender-based violence and promote their protection through psychological, educational, and social support.

## 1. Introduction

### 1.1. Conceptual Definition and Theoretical Framework

The World Health Organization [1] defines violence as the intentional use of physical force or power, whether threatened or actual, against oneself, another person, or a group or community that results in or is likely to result in injury, death, psychological harm, developmental disturbance, or deprivation. Three broad subtypes of violence exist: self-directed, interpersonal, and collective. Thus, gender-based violence is defined as any act of violence committed by a romantic partner, current or former, primarily against women. Gender-based violence is interpersonal violence presented in many areas of life, both public and private. It manifests in the objectification of women’s bodies and in the physical, psychological, social, and sexual abuse that many women experience daily. These forms of violence are detrimental to victims’ physical and mental health, and in extreme cases, they can result in death. Three decades ago, the UN included combating violence against women among its objectives. In Spain, the 2019 Macro-survey on the Prevalence of Gender-based Violence indicates that over half of women over the age of 16 have experienced any form of gender-based violence in their lifetime. Additionally, it is estimated that 1,678,959 children live in households where a woman is a victim of intimate partner violence [2].

Understanding and recognizing violence against women has been a difficult challenge over time because it has been shaped by two fundamental processes, invisibility and naturalization [3,4,5]. The invisibility is linked to the absence of instruments that facilitate its definition and identification, while the naturalization is based on cultural gender stereotypes that influence how we interpret reality [3,4,5]. In this vein, the use of the concept Intimate Partner Violence (IPV), referring to any behavior within a romantic or intimate relationship that causes physical, sexual, or psychological harm, may have hidden this conceptualization based on gender issues. This concept has been widely used in scientific literature to address violence towards women within an intimate relationship. Inn the present study we prefer to use gender-based violence rather than IPV to emphasize gender perspective.

When abused women seek shelter, they usually bring their children with them. Traditionally, the focus has been on protecting and supporting women, which is urgently necessary [6]. However, this focus has resulted in children becoming “silent” or “unidentified” victims of domestic violence. Anderson and Van Ee [6] point out that children are often victims of direct violence in the form of emotional, physical, or sexual abuse by their abusive father. They also note that there is a very limited window in which children evolve from being secondary victims to being directly linked to gender-based violence [7].

In recent decades, there has been a growing interest in the harmful effects of children’s exposure to gender-based violence on their health, learning, and behavior throughout their lives. According to data from nationally representative samples, 11% of children in the United States were exposed to gender-based violence in the past year, and 26% have experienced it at least once in their lifetime. Numerous studies have linked childhood exposure to gender-based violence to diminished physical and mental health as well as behavioral problems throughout one’s life. Among the harmful repercussions linked to exposure to this type of violence in school-aged children are post-traumatic stress disorder (PTSD), internalizing and externalizing behaviors [8].

The bioecological theory of human development [9] provides a better understanding of the negative consequences of gender-based violence exposure in children. According to this theory, children grow up within interconnected environments that influence their development. Research has identified the bidirectional processes through which mothers’ exposure to gender-based violence impacts their children’s mental health [10].

Most studies focus on factors such as education, maternal mental health, attachment, and child temperament as mechanisms linking exposure to gender-based violence to its impact on children. Despite their importance, most research fails to examine whether IPV uniquely impacts child growth compared to other stressors. This is important because various stressors can influence children’s physiology, mental health, and behavior similarly [11]. Some recent studies [8,12] suggest considering physiological and environmental factors to understand the impact of gender-based violence on children’s development. Based on empirical findings and a developmental psychopathology perspective, different trajectories may appear across child development influenced by varying levels of exposure to violence and resilience and self-regulation skills [11]. Three main pathways through which intimate partner violence affect children’s development. These pathways are maternal representations, maternal/fetal physiological stress responses, and maternal mental health. These pathways affect maternal functioning, which influences the child’s parenting behavior and self-regulatory functioning. At the same time, the child develops self-regulatory skills, such as stress response, attachment, and executive functioning, which influence his or her adjustment. Additionally, the bioecological model considers ecological and contextual factors, such as poverty, that affect child development [11].

### 1.2. The Present Study

The present manuscript focused on the psychological consequences suffered by children who were exposed to gender-based violence. The detrimental psychological consequences of gender-based violence manifest not only during childhood and adolescence but also during adulthood, middle age, and old age [13]. A study found that young people affected by this abuse experienced more psychological problems in adulthood, including depression and suicide attempts. They were also more likely to have health and behavioral disorders, such as changes in eating habits, drug use, sexually transmitted infections, and criminal activity. Additionally, individuals who have experienced or witnessed violence within their households tend to have less secure relationships with their partners, struggle to resolve conflicts, and are more likely to become victims or perpetrators of domestic violence [13]. Exposure to gender-based violence during childhood can lead to the development of violent behaviors, thereby increasing the likelihood of engaging in this type of violence in adulthood. According to research by Ehrensaft et al. [14], the primary predictors of this risk are behavioral disorders, exposure to gender-based violence, and power-based punishment systems [15]. Witnessing gender-based violence during childhood profoundly and permanently impacts children’s mental health, affecting their emotional, cognitive, and social development. This exposure can alter their perception of relationships, generate emotional disorders, and increase the risk of developing problematic behaviors in the future.

Despite the importance of this problem in our society, further acknowledgement is still needed for the consequences in children. Two previous reviews have addressed the impact of exposure to IPV on children and youth more than 10 years ago [16,17]. However, an updated review is needed to examine the research evidence in the last decade, with the focus on psychological consequences. Thus, this work aims to perform an updated scoping review to examine the potential psychological consequences that children who were exposed to gender-based violence may develop during childhood and later stages of life. A scoping review was carried out because it is a type of research synthesis that maps the breadth of evidence on a particular topic, identifying key concepts, research gaps, and the types of evidence available. The research question in this scoping review was: What psychological impacts do children exposed to gender-based violence experience? This review also aimed to identify gaps in knowledge and future lines of research and intervention.

## 2. Materials and Methods

### 2.1. Inclusion and Exclusion Criteria

Research published in English and made available via open access between 2015 and 2025 was selected. Only open-access publications were included due to their global accessibility, increased visibility, and greater number of citations, all of which may enhance their societal impact. Other publications may have restrictions on access to the full text, and their scientific and societal impact may be limited for that reason. Open-access publications receive a greater quantity of citations compared to those with accessibility restrictions. To ensure the quality and validity of the information, we included original articles, and excluded theses, conference proceedings, and peer-reviewed websites. The research focuses on children, aged 0–12 years old, who have been exposed to gender-based violence towards their mothers (Population), with no geographic context restriction. Studies prior to 2015 and those that do not directly address gender-based violence or intimate partner violence nor focus on other forms of child abuse were discarded. Due to the growing social awareness of the importance of gender equality, the policies development for the prevention of gender-based violence and reduction in the impact on child development, only publications from the last 10 years have been included. The selected articles should address psychological consequences after exposure to gender-based or intimate partner violence (Concept). No restrictions about context were applied, so that publications from different countries were included, and from different settings, such as clinical, social or educational contexts (Context). No prior review protocol was registered for this scoping review. Table 1 shows inclusion and exclusion criteria.

### 2.2. Literature Search Process

The systematic search of documents carried out on 17 January 2025, was based on the following search string in the used database: “Gender-based violence” OR “domestic violence” OR “intimate partner violence” (title) AND “child*” (title) AND “psychological consequence*” OR symptom* (title). We used quotations to include only articles which examined that specific violence and no other forms and to reduce the number of items that would be discardable for the purpose of the study. Language, year of publication, type of document and open-access restrictions were applied, following the inclusion criteria.

The search was conducted in the following databases: Science Citation Index Expanded, Social Sciences Citation Index, Arts & Humanities Citation Index, Index Chemicus, Current Chemical Reactions, Conference Proceedings: Science, Conference Proceedings: Social Science & Humanities, Grants Index, MEDLINE, ProQuest Dissertations & Theses, Citation Index, SciELO Citation Index, KCI: Korean Journal Database, and Emerging Sources Citation Index. These databases are included in the package of Web of Science, a powerful and multidisciplinary research platform that provides access to a vast collection of academic literature and citation data.

### 2.3. Data Extraction and Data Analysis Procedure

**Figure 1:**
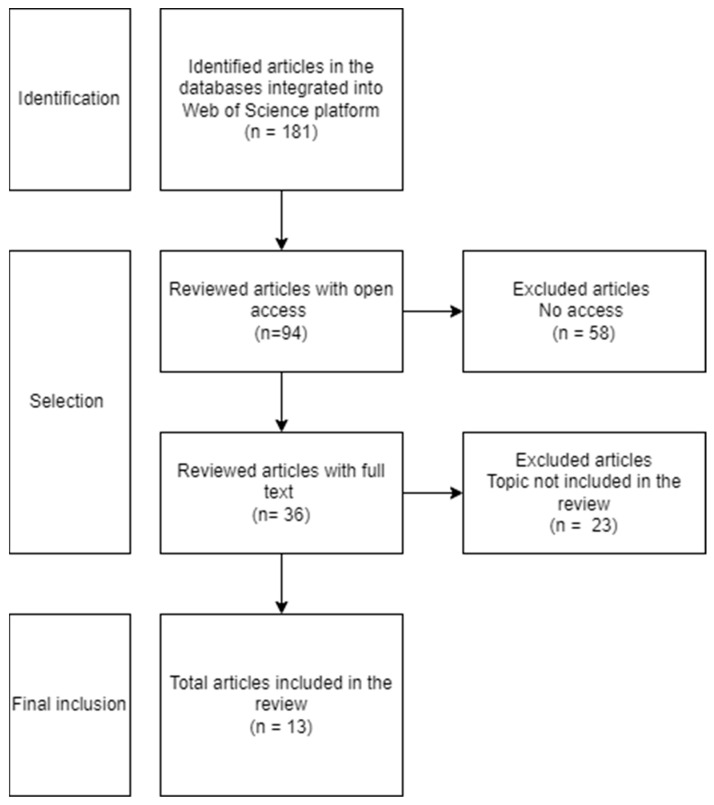
Flow chart.

This scoping review of the literature was based on the PRISMA quality criteria, following the PRISMA Extension for Scoping Reviews (PRISMA-Scr) (see Table A1 in the Appendix A). Figure 1 shows the flow diagram, which illustrates the process of identifying the articles and the results obtained. A full search was conducted on the Web of Science platform, which deleted all duplicates of the results found in the 14 integrated databases. To simplify the search process, all the databases were selected from the beginning of the search on the Web of Science platform.

The two authors independently and blinded to each other’s decisions screened the titles/abstracts and full texts, resolving any conflicts through discussion or a third reviewer. At an initial stage, 181 articles were identified in the databases. Based on titles, 94 articles were selected. Up to 87 articles were excluded at identification stage because they were out of the focus of the review. In the selection stage, 58 articles were excluded because they did not have open access and 23 because they examine a different violence type or sample (n = 23). Finally, 13 documents were selected for inclusion in the review, all of which are scientific articles.

Concerning data analysis procedure, first, content analysis was conducted to examine methodological characteristics of the studies included. A data charting form and list what key information was extracted (e.g., sample, procedure, assessment and results related to psychological effects). Second, a qualitative synthesis based on content analysis of the outcomes was conducted by differentiating internalizing symptoms, externalizing symptoms, post-traumatic stress disorder, post-traumatic stress disorder in mothers and its impact on their children, and long-term consequences. Third, risk of bias and transparent reporting of the articles were analyzed, based on STROBE (Strengthening the Reporting of Observational Studies in Epidemiology). Finally, an analysis of the bibliometric characteristics was performed, by examining impact factors, categories, and citations.

## 3. Results

### 3.1. Content Analysis of the Methodological Characteristics of the Articles

The studies referenced in the articles have been conducted in various parts of the world, as shown in Table 2. Most have been conducted in the United States [10,18,19,20,21,22,23,24]. The rest have been conducted in Spain [25], Australia [7], Sweden [26], Tanzania [27], and China [13]. Regarding sample composition, six of the articles analyzed dyads formed by mothers and children [7,21,22,23,25,27]. Two other studies worked with dyads as well [18,20], but included both mothers and fathers or the children’s caregivers. Four articles focused exclusively on information provided by children and adolescents [10,19,24,26]. One study examined middle-aged and elderly individuals [13]. The ages of the participating minors ranged from one to eighteen years; however, this review will focus on data referring to childhood stage (Table 2). The studies included participants aged between 0 and 12, following the inclusion criteria about exposure in childhood, although some works also examined adolescents [7,10,14,26].

The psychological assessment instruments used are designed to measure specific aspects of emotional, behavioral, and psychological well-being in children, adolescents, and adults. These instruments include tools to evaluate anxiety, depression, trauma, family conflict, violence, stress, and problematic behaviors, among others. These instruments are validated to provide accurate data on mental health and family dynamics. Table 2 shows the instruments used. Scales measuring depression in children included RCMAS [21]; PHQ-9 [24,27]; CES-D [13]; and PAPA [22]. The instruments measuring anxiety were DASS [25], PCL [22], SDQ-P [26], EATQ-R [10], and EQ-P [26]. The instruments measuring posttraumatic stress disorder were IES-R [26], CPSS [7], TSCYC [24,26], TSCC-A [7], and TSI-2-A [7]. The following scales have been found regarding family violence and abuse: CTS [18,20,21]; CTS2 [19,22,25,26]; CTSPC [27]; CDBS [21]; and CREDI [27]. The following scales generally assess child behavior: CBCL [18,19,21,23]; SDQ-P [26]; and YLSI [10]. Finally, scales assessing general psychological disorders, such as the BSI [26] and DASS [25], have also emerged.

As shown in Table 2, six of the studies included have presented a longitudinal design, while the remaining seven have had a cross-sectional design. The longitudinal studies were conducted over periods of six months [21,26], three years [10,19], five years [24], and 25 years [20]. Cross-sectional studies [7,13,18,22,23,25,27] have focused on point measurements, providing insight into the immediate effects of interventions. Table 3 summarizes the design characteristics of the studies.

### 3.2. Content Analysis of the Description of Results

Gender-based violence significantly impacts the mental health and development of children who experience it. According to the reviewed research, exposure to Gender-based violence has been linked to emotional, behavioral, and cognitive changes in children that may occur during or after experiencing Gender-based violence. The most common short-term consequences include internalizing symptoms (i.e., symptoms of depression and anxiety; n = 8) and externalizing problems (i.e., behavioral problems and aggressiveness; n = 11), as well as the onset of post-traumatic stress (n = 5). It should be noted that these symptoms may overlap, and PTSD may present comorbidity with both internalizing and externalizing problems.

#### 3.2.1. Internalizing Symptoms

Internalizing problems such as anxiety (often manifesting as hypervigilance, phobic avoidance, and sleep disturbances) and depression (marked by low self-esteem, loss of interest, and hopelessness) were frequently observed. First, anxiety can manifest in various ways, one of the most common being constant vigilance. Children who have experienced this type of violence tend to be vigilant and overly concerned about the protection and care of themselves or their loved ones. This vigilance can trigger specific phobias and avoidance of places or circumstances that remind them of the violence they experienced [13,20,25].

Second, repeated exposure to this type of violence increases the likelihood that a child will develop depressive symptoms during childhood. These children may exhibit decreased self-esteem, hopelessness, a marked decline in interest in activities they previously enjoyed, and a tendency toward social isolation. This affects their motivation and how they approach daily life [24,25,27].

Third, accumulated fear and anxiety may result in difficulty falling asleep, frequent nighttime awakenings due to intense nightmares about violent events, and difficulty going to bed without an adult present [20,22,23]. Fourth, an emotionally unstable family environment affects a child’s ability to manage his or her own emotions. Many children experience sudden mood swings, alternating between calmness and episodes of uncontrollable crying or panic attacks, what can lead to interpersonal problems [20,27].

#### 3.2.2. Externalizing Symptoms

While internalizing problems primarily affect a child’s emotional well-being, externalizing problems influence how a child interacts with others. These behaviors may include aggressiveness, impulsiveness, and difficulty managing oneself. This increases the likelihood of problems with social and school adaptation [13,20,25]. One significant consequence of exposure to gender-based violence is an increase in aggressive and defiant behaviors toward their siblings, classmates, and caregivers. These behaviors may manifest as physical confrontations, aggressive reactions to disputes, and an increased predisposition to bullying or harassment [7,19,23]. Additionally, children who have experienced gender-based violence often struggle to follow rules and manage their impulses. The absence of positive behavioral patterns and constant stress can result in a lower tolerance for frustration. Consequently, they may respond disproportionately to rules established by adults, exhibiting disobedient or defiant behaviors. Impulsivity may also influence their ability to plan and make decisions [10,26].

Another area affected is school performance. Post-traumatic stress and anxiety can hinder children’s ability to concentrate, which can negatively impact their learning process and school performance [18]. Additionally, it has been found that children who have experienced gender-based violence have low frustration tolerance. Sometimes, this frustration causes them to abandon activities they were initially interested in, out of fear of failing [23,24]. Finally, many children develop resistance to authority. Domestic violence can create a negative perception of authority figures, causing some children to question the rules set by teachers and caregivers. This defiant attitude can hinder their integration into organized settings, such as school, and increase the likelihood of confrontations with adults and peers [18,26].

#### 3.2.3. Post-Traumatic Stress Disorder

In addition to internalizing and externalizing behaviors, exposure to gender-based violence can lead to PTSD, a severe psychological condition that affects a child’s long-term emotional stability. The most common symptoms of PTSD in children who have experienced intimate partner violence include internalizing behaviors such as anxiety, depression, and persistent fear. These children often live in a state of hypervigilance and have a constant perception of insecurity, even in environments that do not pose an immediate threat. Hypervigilance manifests as exaggerated startles and responses to loud noises or certain circumstances. Some children experience sleep disturbances, recurrent nightmares, and intrusive thoughts about the violence they witnessed, which further aggravates their emotional state [7,13,20,22,23]. Another frequent symptom is avoiding memories of the trauma. Some children prefer not to talk about topics related to the violence they experienced or any issue that might cause them to alleviate the trauma. This avoidance, which may be the least painful way for them to deal with the conflict, can lead to emotional numbing or denial of the abuse they suffered, as if it never happened. In more severe cases, dissociative episodes may occur, causing them to withdraw completely from their emotions and reality as a protective mechanism to cope with the pain [7,13,20,23].

#### 3.2.4. Post-Traumatic Stress Disorder in Women and Its Impact on Their Children

Apart from the serious consequences that a child with PTSD may experience, it is also important to consider the consequences for the child when the mother suffers from PTSD. Maternal PTSD impacts not only the mother’s emotional well-being, but also her parenting and, consequently, her children’s development. Women who have experienced gender-based violence are highly likely to suffer from PTSD, which significantly affects their ability to care for and relate to their children. Mothers with PTSD may have difficulty providing protection and emotional support to their children because the disorder can lead to emotional absence or difficulty meeting their children’s needs, especially emotional ones. This can lead to an insecure attachment, where the child does not feel, they can turn to their mother for comfort or protection. This can result in problems with self-esteem and the ability to build healthy relationships in the future.

Additionally, they often resort to rigid or restrictive parenting techniques featuring harsh discipline, such as yelling, severe punishment, and inconsistent rules. In other cases, mothers may have difficulty setting appropriate limits, resulting in disorganized parenting, wherein children lack structure and emotional stability [22]. The age of the child also plays a crucial role in how PTSD develops. For younger children, the emotional bond with their mother is essential. Studies have shown that children of mothers with PTSD are more likely to develop emotional and behavioral disorders, even if they have not witnessed the violence directly. Young children rely on their mothers to assess environmental safety, so if a mother is anxious, constantly alert, or emotionally unstable, her child may internalize these reactions as her own. In contrast, older children tend to develop more independent coping mechanisms, allowing them to better regulate their emotions without relying so heavily on their mother’s psychological state [19,22].

#### 3.2.5. Long-Term Consequences

Examining the long-term consequences of childhood psychological gender-based violence has revealed that its effects persist and intensify during adolescence. This indicates that the impact of such violence accumulates over time, affecting the emotional well-being and coping capacity of minors. Constant exposure to psychological violence often causes children to develop deep distrust of their environment, hindering their ability to form healthy interpersonal relationships and manage stress [18]. Studies have found that individuals who experienced gender-based violence in childhood are at a higher risk for depression in middle and later life. Findings indicate that the greater the frequency of exposure to violence, the higher the depression scale scores, suggesting a cumulative relationship of trauma across the lifespan [24].

Moreover, experiencing gender-based violence during childhood can affect one’s ability to manage stress in adulthood, increasing vulnerability to psychological disorders. Individuals who experienced violence during childhood tend to be more sensitive to stressful situations, which can lead to difficulty managing emotions, problems in interpersonal relationships, and an increased risk of anxiety and persistent depressive disorders [24]. Another crucial factor is the transmission of trauma between generations. Adults who experienced violence during childhood are more likely to exhibit violent behaviors in their romantic or family relationships. This is a consequence of normalizing violence as a form of interaction and lacking effective models for healthy conflict resolution [24].

### 3.3. Risk of Bias and Analysis of Transparent Reporting

Analyzing the bias in the reviewed studies is a fundamental step in evaluating their methodological quality and the validity of their results. This section examines different types of bias (please, see Table 4). Upon evaluating the analyzed studies, it was observed that question formulation bias was controlled, and all studies were classified as green, indicating an adequate definition of research questions [7,10,13,18,19,20,21,22,23,24,25,26,27]. This means that the research questions are well-defined, clear, and concise in all the analyzed studies. Inclusion bias was also satisfactorily addressed in most cases, except for the article by Lv and Li [13], which had slightly defined inclusion criteria.

Several studies marked in yellow exhibit attrition bias, meaning there was a significant loss of participants during the investigation. This could affect the representativeness of the samples and consequently the results [10,14,18,19,21,23,24,25]. Reporting bias occurs when researchers report only certain data and exclude or omit others, which can distort the results. This bias is largely controlled, as several studies appear in green, suggesting that the results were generally reported clearly and transparently. However, some studies still present a risk of bias in communicating their findings [20,23,24,25,26,27].

Furthermore, the following analysis was performed using the STROBE (Strengthening the Reporting of Observational Studies in Epidemiology) table, which is a checklist designed to improve the transparency and quality of observational studies, including cohort, case–control, and cross-sectional studies (please, see Table 5). The selected articles meet most of the STROBE criteria, except for two: a detailed description of the study setting and justification of the sample size. While each article discusses these sections, some lack precise or sufficient information.

### 3.4. Bibliometric Data of Review Documents

The impact factors of the journals in which the articles were published were examined to analyze the relevance of the sources used in this review, as shown in Table 6. The articles belong to journals with impact factors ranging from 0.91 to 13.8. The mean impact factor is 7.35. Jama Network has the highest impact factor, and the Journal of Child and Adolescent Trauma has the lowest.

The quartiles are distributed between Q1 and Q2, indicating the high quality of the selected studies. The journals with the highest quartiles are Child Abuse & Neglect, Development and Psychopathology, JAMA Network, Psychology of Violence, and Journal of Child and Adolescent Trauma. In contrast, the journals with the lowest quartiles are the Journal of Family Psychology, Frontiers in Psychology, Behavioral Sciences, and Child & Family Social Work. Additionally, Greene et al. [22] had the highest number of citations, with a total of 84. This is a considerably higher number than the average of 20.62 found in the chosen articles.

The journal in which the most articles included in this review have been published was Child Abuse & Neglect. Thus, most of the journals included in this review belong to the family studies category, reflecting a focus on analyzing and understanding family dynamics.

## 4. Discussion

Our analysis of the data allows us to conclude that gender-based violence in the family environment has profound and complex consequences for children who witness or experience it [6,8]. Exposure to violence at such a vulnerable stage of life leaves wounds that are not always noticeable at first, but which profoundly affect how children grow and relate to the world [13,25]. The results of the present scoping review are consistent with past review works [16,17]. Short-term mental health problems have been observed, as noted by the review by Artz et al. [16], as well as long-term consequences affecting relationships with peers and future partners, as concluded Wood & Sommers [17]. The present scoping review has provided an updated and detailed overview of the psychological consequences and may underline some research gaps to guide future research.

Consistent with the literature, many children exhibit internalizing symptoms (e.g., anxiety, depression) [24,27], that often go unrecognized by family members and educational professionals [20,22]. Children who remain in a constant state of alertness, experiencing fear in the absence of real threats and losing the ability to enjoy activities they once found pleasurable [10,25]. Furthermore, the persistence of these symptoms even after the violence has ceased suggests a cumulative effect, indicating that emotional damage can endure long after the violent event has occurred [13]. These internalizing symptoms may go unnoticed due to children’s difficulties expressing their emotions and further research would require deeper examination of emotional dysregulation processes and maladaptive cognitive appraisals [28].

Furthermore, the development of externalizing behaviors in these children is considered an obvious indication of their emotional distress [19,23]. Aggressiveness, conduct problems, and disobedience should not be viewed as merely “bad behavior,” but rather as reflections of a chaotic family environment [7,18]. These children often imitate the behavior patterns learned at home in other settings, such as school or with friends [20], and adopted violence as a way to resolve conflicts [26]. These externalizing behaviors as coping strategies to deal with trauma, and may exert a great impact on health, education, and social development across life span [29].

Another concerning aspect is post-traumatic stress disorder (PTSD), which is one of the most severe and long-lasting consequences for children who have experienced intimate partner violence [7,23]. PTSD symptoms such as nightmares, constant vigilance, irrational fear, and intrusive thoughts significantly affect daily life [22,26]. Most alarmingly, these symptoms often occur alongside defiant and aggressive behaviors [20]. These children react in extreme ways due to the constant fear and stress of their environment [19,22]. Their emotional insecurity affects their ability to trust their environment and relate to others in healthy ways [24].

The impact of gender-based violence on children cannot be fully understood without considering the role of the maternal figure [22]. Mothers with PTSD face significant difficulties bonding with their children due to psychological exhaustion, emotional withdrawal, and difficulty establishing clear boundaries [6,27]. This creates an unstable parenting environment that can result in an insecure attachment between mother and child, further increasing the child’s emotional vulnerability [22], which may affect their future social and emotional development [10].

The effects of childhood gender-based violence do not disappear with childhood; they continue into adolescence and adulthood [13,24]. Adolescents who have grown up in violent environments often have low self-esteem, difficulty forming healthy relationships, and a distorted view of conflict [18]. These issues can lead them to perpetuate patterns of violence in their intimate relationships, creating a cycle that is difficult to break [20]. Studies clearly demonstrate that childhood violence leaves a deep mark, affecting not only the present but also how people handle emotional challenges and relationships in adulthood [13,22]. The varied trajectories of internalizing vs. externalizing symptoms underscore the developmental psychopathology idea of multiple pathways of adaptation to trauma [11,30]. Consistently with Bronfenbrenner’s emphasis on the micro-system (e.g., family context and parent–child relationship) in child development, the results underlined the importance of maternal mental health on child development [9,10].

### 4.1. Limitations and Research Gaps Identification

One of the most significant limitations of this study is that it is a literature review and the number of analyzed studies was small. Only open-access publications were included due to their greater accessibility to scientists, professionals, and the community. Future reviews should also include works without open-access and use a greater number of databases. Another limitation of the review comes from the inclusion criteria of selecting publications in the last 10 years. Future review may also address previous publications and integrate evidence from different scientific areas, such as sociology, education and health sciences. Although the article search tried to be exhaustive and detailed, there is still a risk of overlooking relevant knowledge on the subject. Another limitation is that many of the studies analyzed focused mainly on the child’s mother, with little interest in people outside the family, such as teachers, school psychologists, and social workers. It should be noted that future reviews may include publications that are not open access, as well as databases that are not part of the Web of Science. Moreover, future reviews may include grey literature, examining research and information produced outside of traditional academic publishing and distribution channels. Similarly, many of the studies reviewed focused on the maternal figure, neglecting the role of other members of the family. These individuals’ perspectives could have provided a broader, more complete view of the impact of gender violence on minors by addressing contexts such as school and community. Also, many works have followed a cross-sectional design, and more longitudinal studies are needed to examine prospective outcomes across life span. Furthermore, the studies included in this review did not sufficiently address aspects such as sociocultural and economic diversity, which makes it difficult to generalize the conclusions.

Thus, despite the interest of the research evidence included in this review, some areas for future work can be highlighted. Most studies focus on the mother–child context, with little research on other caregivers or community perspectives, and many studies are from high-income countries. Only one study in the review was from an African context (Tanzania) and none from Asia or Latin America. Some underexplored areas have been identified, such as studies involving teachers or wider family, research in diverse cultural and economic contexts, and wide follow-up longitudinal work into adolescence and adulthood. Also, a wide range of consequences may be included, such as those in physical health, educational and labor areas. Few studies examined protective factors to foster resilience after the traumatic experience of gender-based violence exposure, and more evidence is still needed from RCT interventions.

### 4.2. Practical Implications

Despite the difficulties in generalizing the severity of the psychological consequences faced by children due to the limited sample sizes of the examined studies, the importance of the psychological consequences derived from children’s exposure to gender-based violence has been consistently concluded in the studies. Because of these detrimental consequences in child development, some interventions should be fostered to promote resilience after this traumatic experience. Some successful programs have emphasized a resilience approach and the need to offer constructive and compassionate support for recovery. Early interventions focused on trauma-focused therapy, strengthening mother-child attachment, family counseling and school counseling, from a multidisciplinary approach, were found to be protective for child development after this traumatic experience. Two intervention programs that exemplify these characteristics are those conducted by Alanna Foundation in Spain, Refuge Organization in UK, and Child Witness to Violence Project in the US.

The Alanna Foundation is one of the most important programs in Spain addressing the consequences of gender-based violence on children. This nonprofit organization has worked with women who have experienced gender-based violence and their children for more than 20 years [31]. Its network of Integral Care Centers for Women and Children are safe places where children who have experienced such situations can begin the process of emotional healing. The centers employ various professionals, including psychologists, social workers, social educators, and therapists, who work with women and children. They carry out various functions within the program, such as providing psychological care to harmed children and focusing on trauma and resilience. They also carry out educational and school reinforcement activities, so academic performance is affected as little as possible. Additionally, there are play and socialization spaces where children can play and socialize with others in a safe environment. Additionally, family workshops are offered to address the mother-child relationship and promote positive care dynamics, while avoiding the reproduction of violent patterns. Results from this program indicate a noticeable improvement in children’s emotional well-being, with a decrease in symptoms such as anxiety, isolation, and aggressiveness.

An organization in the United Kingdom carries out important tasks related to providing services to victims of gender-based violence. One of the most prominent is called Refuge, an organization that helps thousands of women and children who have experienced gender violence. Its goal is to help these individuals rebuild their lives without fear and minimize the physical, emotional, and economic consequences they experience [32]. Refuge project provides shelters where those affected can safely escape the aggressor. The shelters offer the necessary tools to help them start a new life. A specialized team works with the children, making them feel accompanied, cared for, and valued. These children have spaces to play and develop their social skills while enjoying the company of their peers. Another interesting project is the Child Witness to Violence Project in the United States. This program helps children up to eight years of age who have experienced traumatic events related to gender violence. Since 1992, a team of social workers, educational psychologists, clinical psychologists, and pediatricians have helped thousands of families [33]. The project offers services such as trauma-focused therapy, family counseling, and school counseling. Through these efforts and others, families experience the many benefits of therapy. Caregivers report that children exhibit fewer problem behaviors, express themselves better about the traumatic experience, and that they are more responsive to children’s needs.

## 5. Conclusions

The studies included in this review demonstrate that exposure to gender-based violence during childhood has serious and significant consequences. The findings are based on only 13 studies, which is a limitation to consider when interpreting the findings. Living in a home where this type of violence occurs can trigger symptoms of anxiety, depression, aggressive behavior, and post-traumatic stress disorder. It is important to note that these effects can impact all areas of a child’s life, including the academic, personal, and social spheres, and the possible long-term effects. Also, it is important to emphasize the impact of mental health problems in the mother derived from gender-based violence (i.e., PTSD) on child development. Future research should address the prospective examination of detrimental effects throughout development, the role of other family members, possible moderators in different contexts (e.g., community and school), and cultural and socioeconomic differences. This review emphasizes the importance of treating children as direct victims of gender-based violence and implementing prevention strategies and specific trauma-focused treatments for affected children.

## Figures and Tables

**Table 1:** Inclusion and exclusion criteria.

	Inclusion Criteria	Exclusion Criteria
Date	Studies published between 2015 and 2025	Studies published before 2015
Type of document	Original articles	Books, Theses, Web pages, Conferences, Documents not peer-reviewed
Language	Articles in English	Articles in any language other than English
Accessibility	Open-access studies	Studies with limited access
Population	Children who were exposed to gender-based violence towards their mothers, from birth to adolescence (0–12 years old)	Children who have suffered child abuse, but not gender-based violence Children who have suffered sexual abuse Women who have suffered gender-based violence but have no children
Concept	Psychological consequences	Other consequences
Age	Articles focused on childhood Articles covering other stages, but whose main age focus is childhood	Articles that do not include childhood

**Table 2:** Classification of articles included in the review.

Author (Year)	Sample (Size/Age)	Study Design	Instruments	Key Findings
Capaldi et al. [18]	206 children aged 9 to 10 and their female caregivers. Sample drawn from public schools in Oregon, USA, where juvenile delinquency rates are higher than the city average.	Study examining the main associations between gender-based violence and child abuse with externalizing and internalizing behaviors, academic competence, and social competence	- CBCL - CTS	As child abuse and gender-based violence increase, children’s adjustment in these areas worsens. However, individual abuse has a stronger impact on children than gender-based violence. When they appear at the same time, they influence adolescents more in school performance and children in externalizing behaviors such as aggression and impulsiveness.
Chen [19]	459 children between the ages of 1 and 3. Sample taken in the U.S. from the second National Survey of Child and Adolescent Well-Being.	Longitudinal study examining how child abuse and gender-based violence affect the development of anxious and depressive symptoms, as well as aggressive behavior.	- CBCL - CTSPC - CTS2	The emergence of anxious and depressive symptoms and aggressive behavior among children who have suffered abuse and partner violence demonstrate the negative effects of these
Clark & Hankin [10]	365 children and adolescents between the ages of 7 and 17. Sample drawn from schools in Denver and Colorado	Longitudinal study assessing how exposure to gender-based violence mediates depressive symptoms and self-regulation in those who have experienced it.	- YLSI - EATQ-R	Children who have experienced gender-based violence have lower scores on self-regulation and higher scores on depressive symptoms. In addition to exposure to this type of violence, individual factors also influence scores on these symptoms.
De Oliveira et al. [27]	981 mother-child dyads, aged 18 to 36 months. Sample from the Morongo region of Tanzania.	Study examining the association between gender-based violence, maternal depressive symptoms, harsh child discipline, and child stimulation with child socioemotional development	- PHQ-9 - CTSPC - CREDI	Maternal depressive symptoms may explain the negative association between gender-based violence and children’s socioemotional development. Therefore, clear protocols are needed to help professionals identify this type of violence and make appropriate referrals to protect mothers and children.
Ehrensaft et al. [20]	243 parents and their children, ages 6 to 18. Random sample from 100 different counties in upstate New York.	Longitudinal study that analyzes how children’s exposure to gender-based violence affects them and whether the type of upbringing they receive also influences the symptoms.	- CTS	Children exposed to gender-based violence during childhood are more likely to develop trauma. Intimate partner violence affects parenting, reducing support for the child and increasing negative practices. Positive parenting can act as a protective factor.
Greene et al. [22]	308 mother-child dyads, aged 3 to 6 years. Sample drawn from the Multidimensional Assessment of Preschoolers Study in the U.S., recruited in pediatric clinics.	Study investigating the relationship between post-traumatic stress disorders in mothers who have suffered gender-based violence and the psychopathology of their children	- CTS-2 - PCL - PAPA	Post-traumatic stress disorder experienced by mothers acts as a potential mediator between gender-based violence experienced by mothers and their children’s mental health. They also point to the importance of supporting mothers in their recovery from trauma for their children’s emotional health.
Gower et al. [21]	535 mother-child dyads, ages 7 to 10. Sample drawn from a large urban area in the southern US.	Longitudinal study examining the effects of physical and psychological intimate partner violence on children	- CTS - CPIC-Y - RCMAS - CBCL - CDBS	Intimate partner violence was linked to anxiety symptoms and disruptive behavior in children, even when physical violence was absent. However, when this type of violence occurred, the consequences were more severe.
Lv & Li [13]	10,521 middle-aged and elderly individuals. Sample taken from the China Longitudinal Study of Health and Retirement	Study assessing the relationship between experiencing gender violence during childhood and suffering from depression in middle and old age	- CES-D	Exposure to domestic violence during childhood is associated with a higher likelihood of experiencing depression in middle and old age. Witnessing parental conflict and exposure to corporal punishment were consistently associated with a higher likelihood of experiencing depression later in life.
McDonald et al. [23]	289 mother-child dyads, ages 7 to 12. Sample drawn from community-based domestic violence agencies in Colorado.	Study examining the differential effects of gender-based violence and family contextual factors on children who experience it	- CBCL - CEDV	The findings indicate that environmental factors differentially influence the development of post-traumatic stress disorder and other psychopathological symptoms in children exposed to gender-based violence.
Mertin et al. [7]	50 mother-child dyads, children aged 8–16. Sample drawn from metropolitan domestic violence services in Adelaide, South Australia.	Study that seeks to evaluate maternal and child emotional functioning in relation to post-traumatic stress symptoms in those who have suffered gender-based violence	- CPSS - TSCC-A - TSI-2A	The emotional responses of older children may tend to reflect their own experiences rather than being a reflection of maternal distress, as seems more likely in children who are younger.
Pernebo et al. [26]	50 children aged 4 to 13. Sample taken in Sweden from a mental health service that provides interventions.	Longitudinal study investigating the long-term outcomes of group interventions for children exposed to gender-based violence	- CTS 2 - SDQ-P - TSCYC - EQ-P - BSI - IES-R	Children benefit from interventions and reduce symptoms of gender-based violence. Furthermore, children with more severe trauma symptoms benefited the most from the intervention, although maternal psychological problems may have hindered recovery for some of them.
Ronzón-Tirado et al. [25]	107 mother-child dyads, children aged 6–12. Sample taken from the Comprehensive Monitoring System for Gender-Based Violence in Spain.	A study that analyzes the effects of gender-based violence, adverse experiences after it ends, and the time it takes for depression and anxiety to appear in children.	- CTS2 - DASS	There is a high prevalence of depression and anxiety in children who have experienced gender-based violence. Furthermore, experiences of re-victimization and sustained stress also play a role.
Showalter et al. [24]	580 children between the ages of 3 and 12. Sample taken in the U.S. from a Children’s Advocacy Center.	Longitudinal study examining whether physical abuse and exposure to gender-based violence are associated with depression, anxiety, post-traumatic stress, dissociation, anger, and sexual concerns	- TSCYC - PHQ-9	Physical abuse and gender-based violence are implicated to varying degrees in depression, anxiety, post-traumatic stress, dissociation, anger, and sexual concerns.

**Table 3:** Summary of design characteristics of the studies.

Categories	Classification	Studies
Age range	Only children	Capaldi et al. [18], Chen [19], De Oliveira et al. [27], Greene et al. [22], Gower et al. [21], McDonald & al. [23]), Ronzon-Tirado et al. [25], Showalter et al. [24].
	Children and older	Clark & Hankin [10], Ehrensaft et al. [20], Lv & Li [13], Mertin et al. [7], Pernebo et al. [26]
Context	US	Capaldi et al. [18], Chen [19], Greene et al. [22], Gower et al. [21], McDonald & al. [23], Showalter et al. [24], Clark & Hankin [10], Ehrensaft et al. [20],
	Other countries	De Oliveira et al. [27], Ronzon-Tirado et al. [25], Lv & Li [13], Mertin et al. [7], Pernebo et al. [26]
Data collection procedure	Cross-sectional	Capaldi et al. [18], De Oliveira et al. [27], Greene et al. [22], McDonald & al. [23], Ronzon-Tirado et al. [25], Lv & Li [13], Mertin et al. [7],
	Longitudinal	Chen [19], Gower et al. [21], Showalter et al. [24], Clark & Hankin [10], Ehrensaft et al. [20], Pernebo et al. [26]

**Table 4:** Risk of bias and analysis of transparent reporting of the studies included in the review.

	Question Framing Bias (Review Design Bias)	Inclusion Bias (Inclusion Criteria Bias)	Attrition Bias (Incomplete Outcome Data)	Reporting Bias (Selective Outcome Reporting)
Capaldi et al. [18]	L	L	U	U
Chen [19]	L	L	U	U
Clark & Hankin [10]	L	L	U	U
De Oliveira et al. [27]	L	L	L	L
Ehrensaft et al. [20]	L	L	U	L
Greene et al. [22]	L	L	L	U
Gower et al. [21]	L	L	U	U
Lv & Li [13]	L	U	L	U
McDonald et al. [23]	L	L	U	L
Mertin et al. [7]	L	L	L	L
Pernebo et al. [26]	L	L	L	U
Ronzón-Tirado et al. [25]	L	L	U	L
Showalter et al. [24]	L	L	U	L

Note. High risk of bias: H; Uncertain risk of bias: U; Low risk of bias: L.

**Table 5:** Quality report of the articles.

	Capaldi et al. [18]	Chen [19]	Clark & Hankin [10]	De Oliveira et al. [27]	Ehrensaft et al. [20]	Greene et al. [22]	Gower et al. [21]	Lv & Li [13]	McDonald et al. [23]	Mertin et al. [7]	Pernebo et al. [26]	Ronzón-Tirado et al. [25]	Showalter et al. [24]
Title	L	L	L	L	L	L	L	L	L	L	L	L	L
Abstract	L	L	L	L	L	L	L	L	L	L	L	L	L
Rationale	L	L	L	L	L	L	L	L	L	L	L	L	L
Aim	L	L	L	L	L	L	L	L	L	L	L	L	L
Design	L	L	L	L	L	L	L	L	L	L	L	L	L
Context	L	U	U	L	L	L	U	L	U	L	L	U	L
Sample	L	L	L	L	L	L	L	L	L	L	L	L	L
Variables	L	L	L	L	L	L	L	L	L	L	L	L	L
Sample size	L	L	U	L	L	L	U	L	U	L	U	L	L
Statistical analyses	L	L	L	L	L	L	L	L	L	L	L	L	L
Descriptive data	L	L	L	L	L	L	L	L	L	L	L	L	L
Outcome data	L	L	L	L	L	L	L	L	L	L	L	L	L
Key results	L	L	L	L	L	L	L	L	L	L	L	L	L
Limitations	L	L	L	L	L	L	L	L	L	L	L	L	L
Interpretation	L	L	L	L	L	L	L	L	L	L	L	L	L
Generalization	L	L	L	L	L	L	L	L	L	L	L	L	L

Note: High risk of bias: H; Uncertain risk of bias: U; Low risk of bias: L.

**Table 6:** Bibliometric information of the articles.

Document	Journal	Category	Impact Factor	Quartile	Citations
Capaldi et al. [18]	Child abuse & Neglect	Family Studies	3.4	Q1	12
Chen [19]	Child abuse & Neglect	Family Studies	3.4	Q1	65
Clark & Hankin [10]	Development and Psychopathology	Psychology, Developmental	3.1	Q1	1
De Oliveira et al. [27]	Jama Network	Medicine, General & Internal	13.8	Q1	6
Ehrensaft et al. [20]	Psychology of Violence	Criminology & Penology	2.192	Q1	44
Greene et al. [22]	Child abuse & Neglect	Family Studies	2.845	Q1	84
Gower et al. [21]	Journal of Family Psychology	Family Studies	2.7	Q2	2
Lv & Li [13]	Behavioral Sciences	Psychology, Multidisciplinary	2.5	Q2	2
McDonald et al. [23]	Child abuse & Neglect	Family Studies	2.293	Q1	39
Mertin et al. [7]	Journal of Child and Adolescent Trauma	Family Studies	0.91	Q1	0
Pernebo et al. [26]	Child abuse & Neglect	Family Studies	2.569	Q1	8
Ronzón-Tirado et al. [25]	Frontiers in Psychology	Psychology, Multidisciplinary	2.6	Q2	0
Showalter et al. [24]	Child & Family Social Work	Family Studies	2.386	Q2	5

## Data Availability

The data presented in this study are available on request from the corresponding author due to privacy reasons.

## Appendix A

**Table A1:** Preferred Reporting Items for Systematic reviews and Meta-Analyses extension for Scoping Reviews (PRISMA-ScR) checklist [34].

SECTION	ITEM	PRISMA-ScR CHECKLIST ITEM	REPORTED ON PAGE
TITLE			
Title	1	Identify the report as a scoping review.	1
ABSTRACT			
Structured summary	2	Provide a structured summary that includes (as applicable): background, objectives, eligibility criteria, sources of evidence, charting methods, results, and conclusions that relate to the review questions and objectives.	1
INTRODUCTION			
Rationale	3	Describe the rationale for the review in the context of what is already known. Explain why the review questions/objectives lend themselves to a scoping review approach.	1
Objectives	4	Provide an explicit statement of the questions and objectives being addressed with reference to their key elements (e.g., population or participants, concepts, and context) or other relevant key elements used to conceptualize the review questions and/or objectives.	3
METHODS			
Protocol and registration	5	Indicate whether a review protocol exists; state if and where it can be accessed (e.g., a Web address); and if available, provide registration information, including the registration number.	4
Eligibility criteria	6	Specify characteristics of the sources of evidence used as eligibility criteria (e.g., years considered, language, and publication status), and provide a rationale.	4
Information sources	7	Describe all information sources in the search (e.g., databases with dates of coverage and contact with authors to identify additional sources), as well as the date the most recent search was executed.	4
Search	8	Present the full electronic search strategy for at least 1 database, including any limits used, such that it could be repeated.	4
Selection of sources of evidence	9	State the process for selecting sources of evidence (i.e., screening and eligibility) included in the scoping review.	5
Data charting process	10	Describe the methods of charting data from the included sources of evidence (e.g., calibrated forms or forms that have been tested by the team before their use, and whether data charting was done independently or in duplicate) and any processes for obtaining and confirming data from investigators.	Not Applicable
Data items	11	List and define all variables for which data were sought and any assumptions and simplifications made.	Not Applicable
Critical appraisal of individual sources of evidence	12	If done, provide a rationale for conducting a critical appraisal of included sources of evidence; describe the methods used and how this information was used in any data synthesis (if appropriate).	5
Synthesis of results	13	Describe the methods of handling and summarizing the data that were charted.	5
RESULTS			
Selection of sources of evidence	14	Give numbers of sources of evidence screened, assessed for eligibility, and included in the review, with reasons for exclusions at each stage, ideally using a flow diagram.	6
Characteristics of sources of evidence	15	For each source of evidence, present characteristics for which data were charted and provide the citations.	7
Critical appraisal within sources of evidence	16	If done, present data on critical appraisal of included sources of evidence (see item 12).	13
Results of individual sources of evidence	17	For each included source of evidence, present the relevant data that were charted that relate to the review questions and objectives.	7
Synthesis of results	18	Summarize and/or present the charting results as they relate to the review questions and objectives.	10
DISCUSSION			
Summary of evidence	19	Summarize the main results (including an overview of concepts, themes, and types of evidence available), link to the review questions and objectives, and consider the relevance to key groups.	15
Limitations	20	Discuss the limitations of the scoping review process.	16
Conclusions	21	Provide a general interpretation of the results with respect to the review questions and objectives, as well as potential implications and/or next steps.	18
FUNDING			
Funding	22	Describe sources of funding for the included sources of evidence, as well as sources of funding for the scoping review. Describe the role of the funders of the scoping review.	18
